# Therapeutic Potential of a Novel *Stenotrophomonas maltophilia* Phage XAN_XB1: Isolation, Characterization, Genome Analysis and Evaluation in Mice Model

**DOI:** 10.3390/ijms27020944

**Published:** 2026-01-18

**Authors:** Qingqing Yang, Baoyu Gan, Zhonglin Wang, Shan Jiang, Cao Qiu, Yawen Wang, Bing Liu, Xiaoyan Zeng

**Affiliations:** 1 Department of Laboratory Medicine, The First Affiliated Hospital of Xi’an Jiaotong University, Xi’an 710061, China; yangqingqing@xjtufh.edu.cn (Q.Y.); wangzhonglin@xjtufh.edu.cn (Z.W.); jiangshan@xjtufh.edu.cn (S.J.); 2 BioBank, The First Affiliated Hospital of Xi’an Jiaotong University, Xi’an 710061, China; ganbaoyu@xjtufh.edu.cn (B.G.); wangyw1269@mail.xjtu.edu.cn (Y.W.); 3 State Key Laboratory for Diagnosis and Treatment of Severe Zoonotic Infectious Diseases, Key Laboratory for Zoonosis Research of the Ministry of Education, Institute of Zoonosis, College of Veterinary Medicine, Jilin University, Changchun 130062, China; qiucao24@mails.jlu.edu.cn

**Keywords:** *Stenotrophomonas maltophilia*, bacteriophage, drug-resistant bacteria, phage therapy

## Abstract

A novel lytic bacteriophage, XAN_XB1, was isolated from hospital wastewater through host bacterial enrichment and evaluated for its potential in controlling multidrug-resistant *Stenotrophomonas maltophilia* infections. Transmission electron microscopy revealed that XAN_XB1 has a long tail, possessing an icosahedral head of ~80 nm in diameter and a tail measuring ~150 nm in length. It produced clear plaques of 0.5–1 mm on host bacterial lawns. Host range analysis demonstrated its ability to infect multiple multidrug-resistant *S. maltophilia* isolates. Biological characterization showed that the phage is chloroform-insensitive, retains strong lytic activity across a wide temperature (4–60 °C) and pH (3.0–10.0) range, and achieves more rapid host suppression under higher multiplicity of infection (MOI). Whole-genome sequencing determined a ~47 kb double-stranded DNA genome encoding 71 predicted open reading frames, with no known virulence or antibiotic resistance genes. Phylogenetic analysis of MCP and terminase large subunit sequences placed XAN_XB1 in a unique *Caudoviricetes*, with ANI values below the 95% ICTV threshold verifying its status as a novel phage species. The XAN_XB1 therapy significantly alleviates *S. maltophilia* infection-induced severe pulmonary inflammatory lesions, high mortality, elevated serum inflammatory factors and massive pulmonary bacterial colonization in male BALB/c mice, confirming its favorable therapeutic effect on such infections. Collectively, these results reveal that is an efficacious candidate for therapeutic development against *S. maltophilia* infections.

## 1. Introduction

Antimicrobial resistance (AMR) has escalated into one of the most critical challenges to global public health [1,2]. While antibiotics have long been the cornerstone of infectious disease treatment, their extensive misuse and overuse have accelerated the spread of AMR pathogens and the rise of multidrug-resistant (MDR) bacteria [3,4]. These organisms are especially concerning in clinical settings, where they often cause severe hospital-acquired infections in immunocompromised patients and are associated with high morbidity and mortality. Among these MDR bacteria, *Stenotrophomonas maltophilia* has gained increasing attention. This Gram-negative, non-fermenting bacterium is widely distributed in the environment but has become a significant opportunistic pathogen in hospitals. Clinically, it is now the third most frequently isolated non-fermenting Gram-negative bacillus, after *Acinetobacter* spp. and *Pseudomonas aeruginosa* [5]. Mortality rates from *S. maltophilia* infections can range from 21% to 69% [6]. Although not considered highly virulent, it is a major cause of iatrogenic infections, especially in immunocompromised individuals, long-term hospitalized patients, and those with chronic respiratory disease or undergoing invasive medical procedures [7,8,9]. Its clinical detection rate continues to rise worldwide [10], highlighting its growing importance as a nosocomial pathogen.

Clinical treatment of *S. maltophilia* infections faces particularly challenging, primarily due to the bacterium’s extensive intrinsic resistance to multiple antibiotics. This resistance stems from four core mechanisms: constitutive production of L1 metallo-β-lactamase and L2 serine β-lactamase, an active multidrug efflux pump system, low outer membrane permeability, and a complex metabolic regulatory network [9]. Consequently, *S. maltophilia* exhibits resistance to carbapenems (e.g., imipenem, meropenem), most β-lactams, and aminoglycosides [11,12]. Limited by this multidrug resistance, effective clinical treatment options are extremely scarce. Currently, trimethoprim-sulfamethoxazole (SXT) remains the first-line therapy, with fluoroquinolones and tetracyclines as alternative agents—all treatments must be strictly guided by antimicrobial susceptibility testing results [9,13]. Furthermore, the high genetic diversity of *S. maltophilia* complicates clinical infection management [8], collectively underscoring the urgency of developing novel therapeutic strategies.

Against this backdrop, phage-mediated targeted therapy has emerged as a research hotspot. Although phage therapy has not yet been incorporated into globally unified, standardized treatment guidelines, its clinical application is gaining increasing formal recognition and normative guidance from authoritative bodies. For instance, the European society has issued consensus statements endorsing its use for multidrug-resistant (MDR) infections with no alternative therapies, and the U.S. FDA has provided a regulatory pathway through mechanisms such as the Investigational New Drug (IND) application [14,15]. China has recently released an expert consensus to standardize its clinical application management [16]. Collectively, these documents form the current reference framework for implementing phage therapy.

The status of *S. maltophilia* as a specific niche bacterium for phages stems from the synergistic effects of its ecological and molecular characteristics, which have fostered a unique coevolutionary relationship with phages—distinguishing it from most phage–host systems. Ecologically, *S. maltophilia* is widely distributed in natural environments and clinical settings [17], providing a stable and extensive reservoir for phage contact and survival, which lays the foundation for phage enrichment. At the molecular level, phages must parasitize in living cells and exhibit host specificity, which primarily depends on phage receptor-binding proteins (RBPs) and bacterial surface receptors [18]. The specificity between *S. maltophilia* and phages is primarily rooted in the precise matching between phage RBPs and bacterial surface receptors, particularly type IV pili [19], and is synergistically regulated by the inherent drug-resistant barriers of the bacterium and the coevolutionary relationship between the two. The drug-resistant structures of this bacterium, such as low outer membrane permeability and multidrug efflux pumps, form a “molecular sieve” that not only filters out broad-host-range phages but also reversely drives the evolution of highly specific phages with adaptive infection mechanisms, ultimately establishing an exclusive niche relationship where phages only infect specific bacterial strains [20]. This specificity not only makes *S. maltophilia* an excellent model for studying phage-bacteria coevolution but also lays a foundation for the development of precision phage therapy.

As of 2025, a total of 86 phages targeting *S. maltophilia* have been discovered and characterized (www.ncbi.nlm.nih.gov). Notable examples include DLP1–DLP6 [21], AXL-3 [22], BUCT603 [20], CUB19 [23], and vB_SmaP_c9-N [13]—have been reported, demonstrating strong bactericidal activity and host specificity. Nevertheless, research into the taxonomic diversity, biological properties, genomic features and therapy of *S. maltophilia* phages remains limited. To address this gap, the present study reports the isolation and characterization of a novel lytic phage, tentatively named XAN_XB1, from hospital wastewater using *S. maltophilia* as the host. Its biological characteristics, including multiplicity of infection (MOI), killing curve, one-step growth dynamics, stability across temperature and pH, host range, and morphology, were systematically evaluated. In addition, whole-genome sequencing and annotation were conducted to assess its genetic features and therapeutic potential. Moreover, a murine infection model was established to assess the in vivo therapeutic potential of XAN_XB1 against *S. maltophilia*-induced infections, providing direct experimental evidence for its clinical applicability.

## 2. Results

### 2.1. Isolation and Purification of Phage XAN_XB1

**Figure 1:**
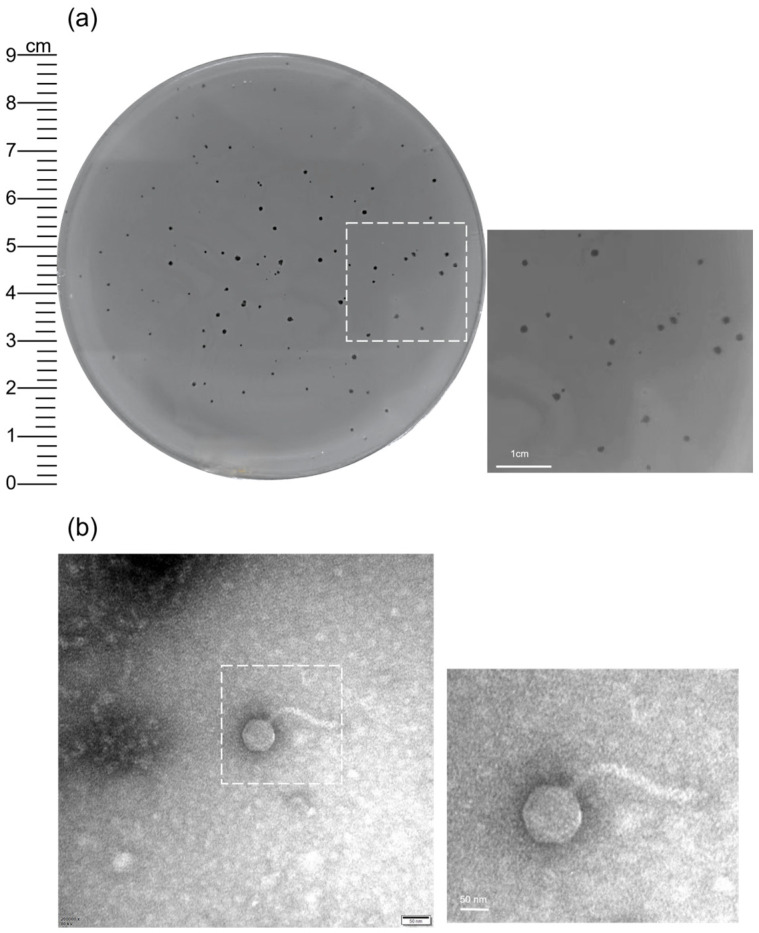
Isolation and characterization of phage XAN_XB1. (**a**) Plaque morphology of phage XAN_XB1 on *S. maltophilia* was characterized by clear, round shapes with diameters of 1.0–1.5 mm, as visualized by the double-layer agar method. Scale bar: 1 cm. (**b**) Transmission electron microscopy (TEM) image of phage XAN_XB1, showing an icosahedral head and a long, non-contractile tail. Scale bar: 50 nm.

Phages infecting *S. maltophilia* were isolated from hospital wastewater collected from multicenter hospitals in Northwest China using the double-layer agar method. The clinical strain *S. maltophilia* No.200019 (resistant to Levofloxacin and SXT) served as the indicator host. After three rounds of plaque purification, a single lytic phage was obtained and designated XAN_XB1. It formed clear, round plaques with diameters of 1.0–1.5 mm on bacterial lawns (Figure 1a).

### 2.2. Morphological Characteristics of XAN_XB1

TEM showed that XAN_XB1 possesses an isometric icosahedral head with a diameter of 80 ± 3 nm and a long, flexible, non-contractile tail measuring 150 ± 5 nm (*n* = 3, Figure 1b); thus, the virion exhibits *Siphovirus*-like morphology.

### 2.3. Host Range Assessment

**Figure 2:**
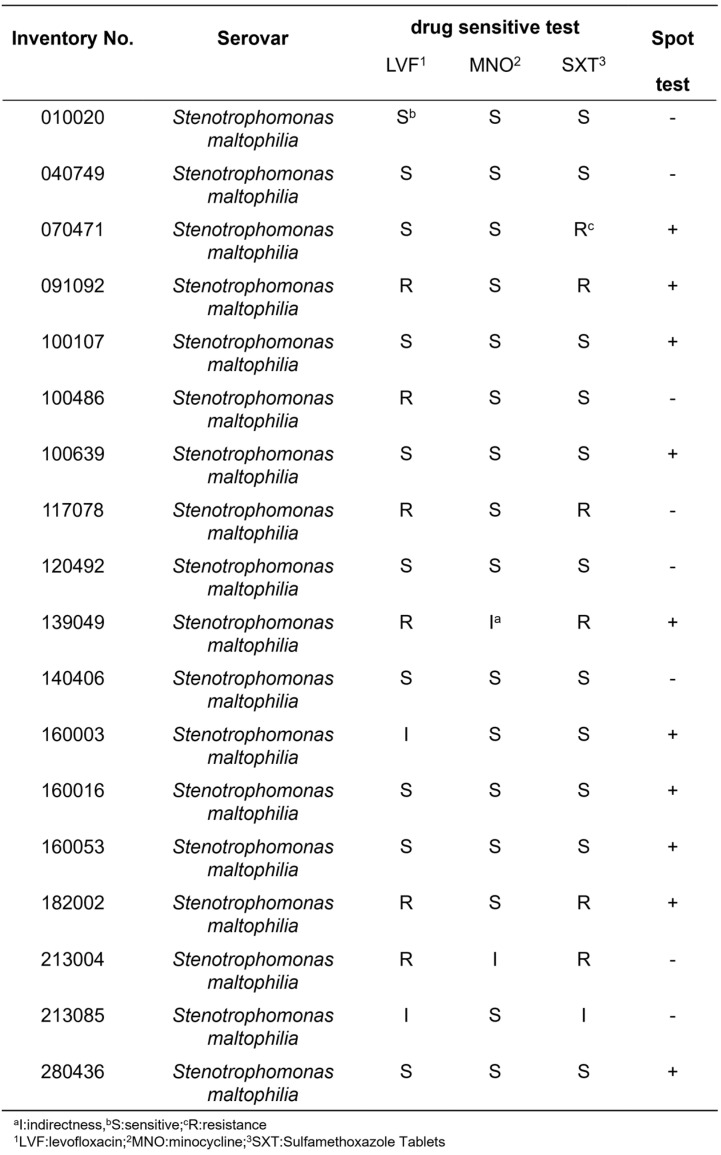
Host range analysis of phage XAN_XB1 against 18 clinical isolates of *S. maltophilia*.

The host range was assessed using spot assays against 18 clinical isolates of *S. maltophilia* (5 multidrug-resistant and 13 drug-sensitive). XAN_XB1 lysed 10 strains (55.6%), including 3 multidrug-resistant isolates (Figure 2), demonstrating a relatively broad host range.

### 2.4. Genomic Features of XAN_XB1

**Figure 3:**
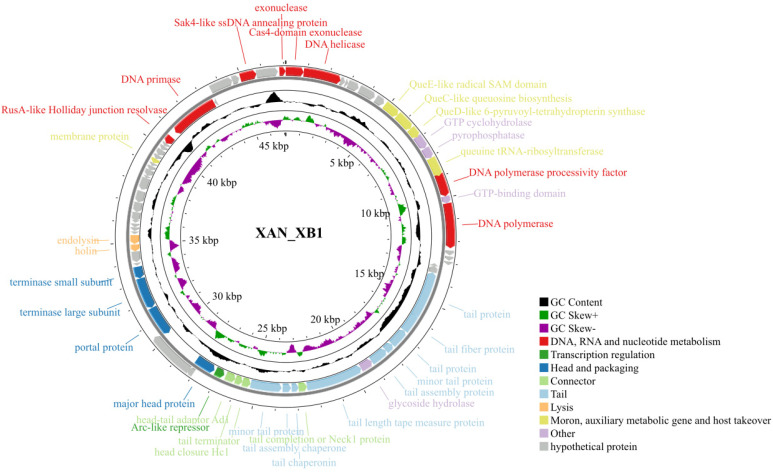
Genomic analysis of phage XAN_XB1. Circular genome map of XAN_XB1 generated using Proksee. Predicted ORFs are indicated by arrows according to transcriptional orientation. ORFs with putative functions are color-coded by functional category, while hypothetical proteins are not shown. The outer ring represents coding regions, and the inner rings depict GC content and GC skew profiles.

**Figure 4:**
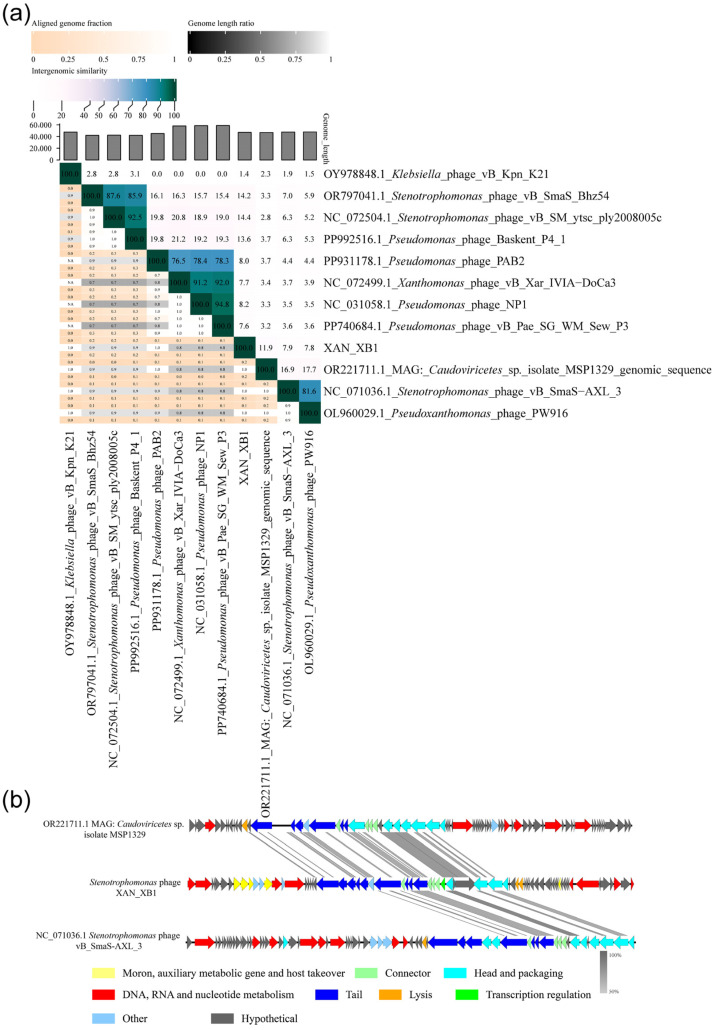
Whole-genome similarity analyses and collinearity, functional module, of phage XAN_XB1 and reference phages. (**a**) Whole-genome similarity heatmap of XAN_XB1 and reference phages. (**b**) Collinearity and functional module analysis of phage XAN_XB1 and reference phage genomes.

**Figure 5:**
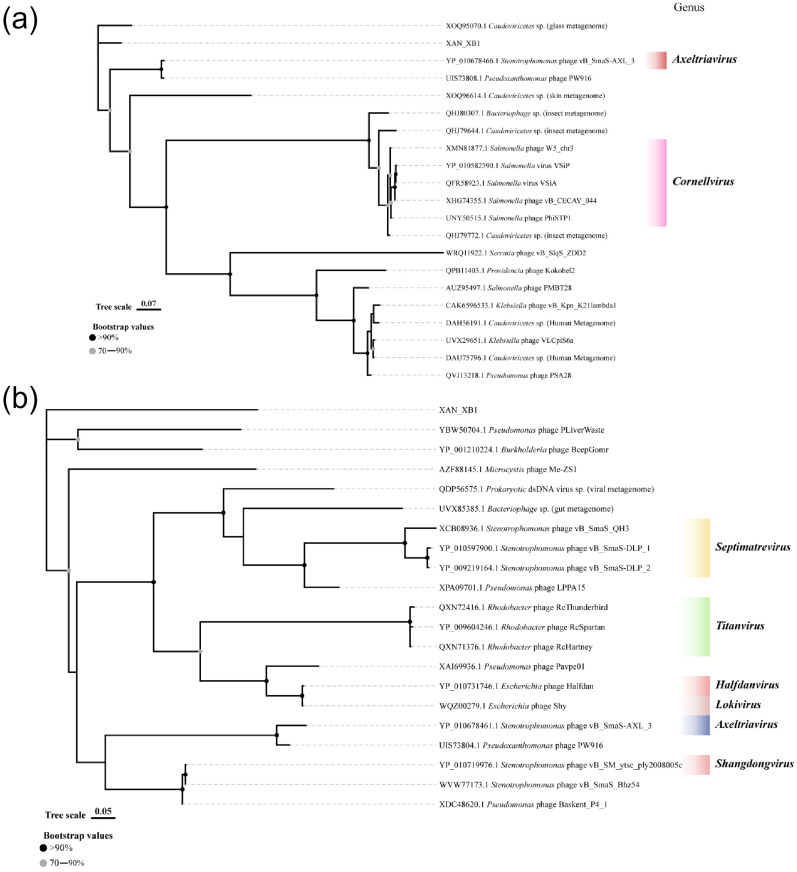
Phylogenetic tree of phages based on MCP and large terminase subunit sequences. (**a**) Phylogenetic tree of *Caudoviricetes taxa* based on MCP sequences. (**b**) Phylogenetic tree of phages based on large terminase subunit sequences.

Whole-genome sequencing using Illumina NovaSeq (Illumina, Inc., San Diego, CA, USA) yielded a linear double-stranded DNA genome of 47,092 bp with a GC content of 58.4%, which is a little lower than that of its host (66.47%). A total of 71 open reading frames (ORFs) were predicted by Pharokka, and then were functionally annotated by BLASTp v2.17.0 and HHpred(version: 57c8707149031cc9f8edceba362c71a3762bdbf8). These included genes associated with DNA/RNA and nucleotide metabolism, transcription regulation, head and packaging, connector, tail, lysis, moron, auxiliary metabolic gene and host takeover. The genome encodes several genes putatively involved in nucleotide modifications. No tRNA genes, lysogeny-related genes, virulence factors, or antibiotic resistance genes were identified, supporting its strictly lytic lifestyle (Figure 3). From the VIRIDIC heatmap, the average nucleotide identity (ANI) between XAN_XB1 and its closest relative, *Caudoviricetes* sp. isolate MSP1329, was determined to be 11.9%. ANI values between XAN_XB1 and all other related phages were consistently below 10% (Figure 4a). As shown in Figure 4b, XAN_XB1 exhibited significant differences in genomic synteny and functional module distribution compared with two phages, with no obvious collinear regions observed between the two phages. These results collectively confirm that XAN_XB1 is a novel *Stenotrophomonas* phage. Phylogenetic analysis based on the major capsid protein (MCP) (Figure 5a) and terminase large subunit sequence (Figure 5b) confirmed XAN_XB1 belongs to *Caudoviricetes* but forms an independent lineage, with no clustering to known families or genera. This is consistent with its low intergenomic similarity and distinct genomic collinearity. All above collectively support the formal classification of XAN_XB1 within the class *Caudoviricetes*; notably, our comprehensive analyses suggest that this phage does not fit any currently ICTV-recognized family, subfamily, or genus and may represent a novel taxonomic group within *Caudoviricetes* [24].

### 2.5. Stability Under Different Physicochemical Conditions

**Figure 6:**
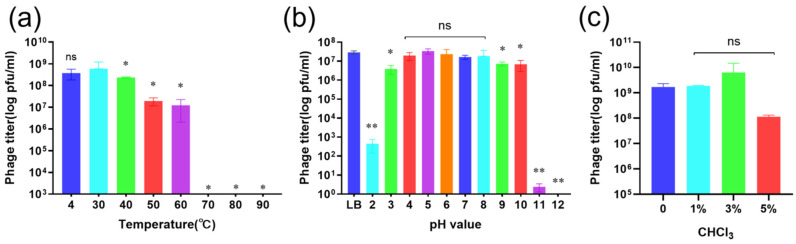
Stability profile of phage XAN_XB1 under various physicochemical conditions. (**a**) Thermal stability was evaluated by incubating phage suspensions at temperatures ranging from 4 °C to 90 °C for 1 h. (**b**) pH stability of XAN_XB1 was assessed by incubating phage suspensions (~10^8^ PFU/mL) in buffers spanning pH 2–12 for 1 h at room temperature. (**c**) Chloroform sensitivity was determined by treating phage suspensions with chloroform concentrations up to 5% (*v*/*v*) for 30 min. In all assays, residual phage titers were quantified by plaque assay. Data represent the mean of three independent biological replicates, each with three technical replicates. * *p* < 0.05, ** *p* < 0.01.

XAN_XB1 retained >90% activity after incubation at 4–50 °C for 1 h but showed reduced infectivity (<10%) at 60 °C and complete inactivation at 70 °C (Figure 6a). The phage exhibited robust stability, retaining over 90% of its activity within the pH range of 3.0–10.0, with significant loss of infectivity below pH 2.0 or above pH 11.0 (Figure 6b). Exposure to chloroform at 1–5% (*v*/*v*) for 30 min had no significant effect on phage titer (*p* > 0.05), confirming resistance to lipid solvents (Figure 6c).

### 2.6. One-Step Growth Curve and Optimal MOI

**Figure 7:**
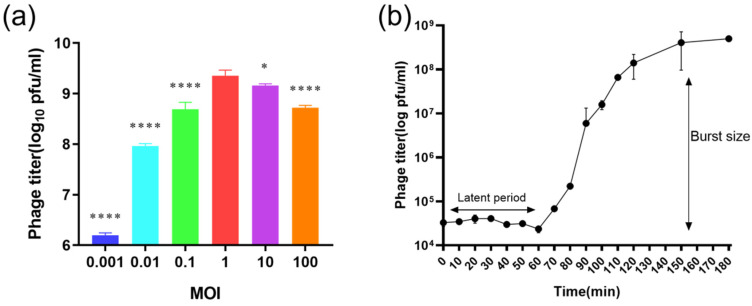
Characterization of phage XAN_XB1 infection dynamics. (**a**) Analysis of the MOI for phage XAN_XB1. Multiple MOIs ranging from 0.001 to 100 were tested to evaluate phage productivity after infection of *S. maltophilia* at 37 °C. (**b**) One-step growth curve of XAN_XB1 showing the latent period and burst phase following infection at the optimal MOI. Phage titers were measured at regular intervals by plaque assay. Values shown represent the mean of three independent experiments. * *p* < 0.05, **** *p* < 0.0001.

Optimal multiplicity of infection (MOI) determination showed the highest titer (2.3 × 10^9^ PFU/mL) at an MOI of 1, which was selected for subsequent assays (Figure 7a). The one-step growth curve demonstrated a latent period of 60 ± 3 min, followed by a burst period of 100 ± 5 min. The average burst size was estimated at 228 ± 15 PFU per cell (Figure 7b).

### 2.7. XAN_XB1 Exerted a Potent Therapeutic Effect Against S. maltophilia Infection in Mice

**Figure 8:**
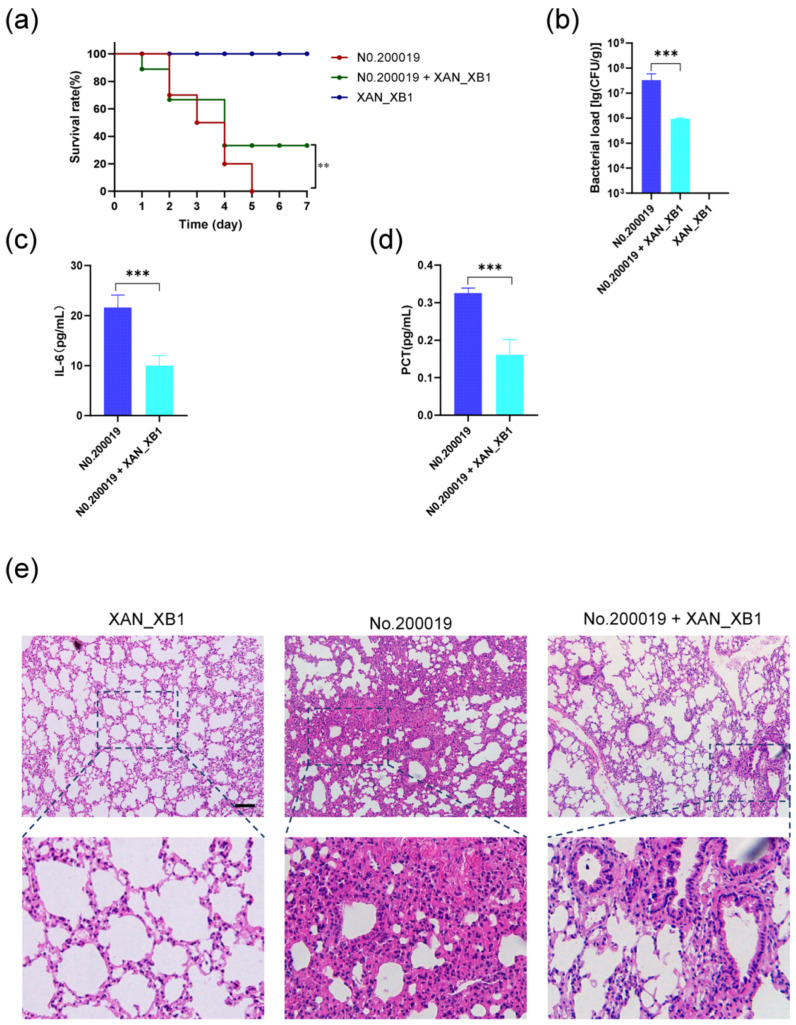
Assessment of phage XAN_XB1 efficacy following infection in mice. (**a**) Monitoring of survival in infected mice after intervention of phage XAN_XB1. (**b**) Lung bacterial load of mice. (**c**) Serum level of IL-6 of mice. (**d**) Serum level of PCT of mice. (**e**) The H&E stained image of mouse lung tissues. Scale bar: 100 μm. *** *p* < 0.001.

Complete mortality was observed in the positive control group by 5 days post-infection, in stark contrast to the negative control group, where all mice survived until the end of the experiment. In phage XAN_XB1-treated mice with bacterial infection, early-phase mortalities (days 1–4 post-infection) were noted, yet the survival rate stabilized and did not decline further after day 4. Phage treatment significantly enhanced the 7-day survival rate of infected mice by 30% (*p* < 0.01; Figure 8a). This survival benefit was accompanied by a sharp reduction in pulmonary bacterial burden. At 30 h post-treatment, the bacterial load reached 10^7^ CFU/g in the positive control group, whereas phage XAN_XB1 therapy sharply reduced it to approximately 10^5^ CFU/g (*p* < 0.001; Figure 8b). In the phage-treated group, serum levels of inflammatory markers were significantly reduced compared to the infection control group. Specifically, the concentration of interleukin-6 (IL-6) in the serum of treated mice decreased to 9.98 ± 0.87 pg/mL at 30 h post-treatment, which was markedly lower than that in the infection control group (21.6 ± 0.64) pg/mL (*p* < 0.001) (Figure 8c). Similarly, the serum procalcitonin (PCT) level also showed a notable decline following phage intervention, with the treatment group measuring (0.1608 ± 1.1) pg/mL compared to (0.3252 ± 0.78) pg/mL in the infection control group (*p* < 0.001) (Figure 8d). In light of these findings, that phage therapy effectively alleviates the systemic inflammatory response induced by *S. maltophilia* infection. Histopathological examination further confirmed that the lungs of mice infected with *S. maltophilia* exhibited severe inflammation, characterized by markedly widened alveolar septa, diffuse infiltration of a large number of inflammatory cells, and obvious compression and deformation of alveolar lumens due to interstitial hyperplasia and inflammatory infiltration, with inflammation involving most areas of the lung tissue. In contrast, phage XAN_XB1 treatment resulted in moderate inflammation, as evidenced by mild-to-moderate widening of alveolar septa, focal infiltration of inflammatory cells, slight morphological changes in alveolar structures, and a significant alleviation of overall inflammation (Figure 8e).

## 3. Discussion

The escalating prevalence of multidrug-resistant *S. maltophilia* infections, combined with the scarcity of effective antibiotics, necessitates alternative therapeutic approaches. There exists significant genetic and phenotypic heterogeneity within the *S. maltophilia* genus, enabling these bacteria to rapidly adapt to changing selective pressures in both clinical and environmental settings [17,25,26,27]. This high level of genetic diversity is even observable among isolates from the same hospital [28], and clinical isolates exhibit a higher mutational frequency than more prone to developing drug resistance than environmental isolates, posing greater challenges for clinical treatment. In this study, we obtained isolated strains from clinical sources and isolated a previously unreported bacteriophage from hospital wastewater. Herein, we detail the isolation and characterization of the novel lytic phage, XAN_XB1, with several properties that highlight its potential for therapeutic application. Morphologically, XAN_XB1 has a long, non-contractile tail, consistent with many Gram-negative bacteriophages. Its structural features, including a long non-contractile tail, are typically associated with host recognition and specificity. The formation of clear plaques (1.0–1.5 mm) reflects efficient bacterial lysis, a favorable trait for therapeutic use.

Genomic analysis confirmed the safety of XAN_XB1 for therapeutic use. Its genome lacks lysogeny-associated genes, virulence factors, and antibiotic resistance determinants, confirming a strictly lytic lifestyle and minimizing the risk of horizontal gene transfer. VIRIDIC analysis demonstrated low intergenomic similarity (<20%) between XAN_XB1 and other phages, and subsequent Easyfig analysis revealed only minimal grey-line sequence matches but no substantial collinear regions. Comprehensive phylogenetic analysis of MCP and large terminase subunit clearly defined the taxonomic status of XAN_XB1, it belongs to the *Caudoviricetes*; MCP-based analysis only supports its placement in the *Caudoviricetes*, while large terminase subunit-based analysis shows it forms an independent clade with unique sequences; thus, it is identified as a novel species of *Caudoviricetes*. Comparison with previously described phages such as BUCT603 [20], CUB19 [23], and vB_SmaP_c9-N [29] indicates both shared and unique traits. While all exhibit strong lytic activity, XAN_XB1 demonstrates enhanced stability and broader host range, which may provide an advantage for clinical use. A particularly important finding is the relatively broad host range of XAN_XB1, with lytic activity against more than half of the tested clinical isolates, including multidrug-resistant strains. Although SXT susceptibility is still common, resistance phenotypes are becoming more frequent [30]. The XAN_XB1, isolated in this work, exhibited a broad lytic spectrum, encompassing most of the SXT-resistant clinical strains tested. This indicates that the phage’s lytic mechanism is independent of the primary SXT resistance pathways, warranting further investigation into its receptor specificity. This breadth increases its potential utility in diverse clinical scenarios where *S. maltophilia* strain heterogeneity complicates treatment. Expanding phage libraries is essential, as no single phage will be sufficient to cover the genetic diversity of *S. maltophilia* isolates encountered in practice. pH and temperature tolerance are critical prerequisites for the clinical translation of bacteriophages. The XAN_XB1 demonstrates superior stability compared to previously reported phages within the same genus through systematic comparison. In terms of pH stability, the phage maintained a PFU retention rate of over 85% after incubation for 1 h across a pH range of 3.0 to 10.0. In contrast, the phage BUCT603, DLP1, DLP2 experienced a sharp decline in activity at pH < 4.0 [20,31]. This difference indicates that the new phage possesses a greater ability to withstand the pH fluctuations present in complex clinical bodily fluids, offering potential for diverse administration routes such as oral delivery or pulmonary aerosolization. Regarding thermal stability, the XAN_XB1 retained over 90% of its activity after storage at 4–50 °C, and retained over 60% of its activity even at 60 °C, outperforming some phages [20,32], which enables it to remain effective in high-temperature conditions. Such stability suggests robustness under both storage and physiological conditions, enhancing its therapeutic applicability. The optimal MOI of 1 and large burst size (228 PFU/cell) further support its strong replicative capacity and rapid bacterial killing potential.

*S. maltophilia* infection can cause severe pulmonary inflammatory lesions in mice, manifested as extensive diffuse inflammatory cell infiltration, significant widening of alveolar septa and, obvious damage to alveolar structures, high mortality rate, elevated serum inflammatory factors and massive bacterial colonization in lung tissue. The majority of newly isolated *S. maltophilia* phages have not undergone animal testing to evaluate their safety and therapeutic potential [21,22,33,34]. In contrast, this study conducted in-depth preclinical animal studies on the newly isolated XAN_XB1. We not only validated its significant in vivo protective efficacy in a murine lung infection model but also systematically assessed its safety and immunogenic response through tissue bacterial load analysis, inflammatory cytokine profiling, and histopathological examination. This study indicates that XAN_XB1 therapy can significantly alleviate the above pathological changes in lung tissue, reduce the degree and scope of inflammation, and lower the mortality rate of infected mice, down regulate the levels of serum inflammatory cytokines, and effectively reduce the pulmonary bacterial load, thereby protecting the integrity of lung parenchyma. These data provide, for the first time, a comprehensive demonstration of the therapeutic potential and biosafety of XAN_XB1 at the animal level, marking a crucial step in its transition from in vitro research toward clinical translation and application. This study comprehensively confirms the good therapeutic effect of phage on pulmonary infection caused by *S. maltophilia* from multiple dimensions, which provides a valuable experimental reference for the further development and clinical application of phage therapeutic preparations. Limitations should be acknowledged, such as reliance on a single animal model, absence of long-term follow-up studies, narrow host range evaluation, and incomplete genomic annotation. Future research should optimize experimental models, expand clinical strain panels, and employ multi-omics technologies, develop phage cocktails, and explore synergy with antibiotics to reinforce conclusions and facilitate clinical translation.

## 4. Materials and Methods

### 4.1. Bacterial Isolates and Cultivation Media

The *S. maltophilia* strain (isolated from The First Affiliated Hospital of Xi’an Jiaotong University) used in this study was a clinically isolated drug-resistant strain, employed for phage screening. Additionally, 18 clinically isolated strains were selected to delineate the host range of the newly isolated phage. All strains were cultured in Luria–Bertani (LB) (AOBOXING BIO-TEC Co., Ltd., Beijing, China) liquid medium or on 1.5% LB agar plates at 37 °C, and stored at −80 °C in 50% glycerol (Sinopharm Chemical Reagent Co., Ltd., Shanghai, China). Bacterial susceptibility to antimicrobial agents was evaluated via either the disk diffusion assay or E-test strips (Wenzhou Kangtai Biotechnology Co., Ltd., Wenzhou, China), following standard clinical laboratory protocols.

### 4.2. Phage Isolation and Purification

A novel phage infecting *S. maltophilia* was isolated from untreated mixed wastewater samples collected from the sewage systems of multiple hospitals. Using *S. maltophilia* strain 200019 as the host, phages were enriched directly from wastewater and isolated via the double-layer agar method. Briefly, untreated wastewater samples were centrifuged and filtered through a 0.22 μm filter (MilliporeSigma, Burlington, MA, USA) to remove bacteria and other particles. Mixing 500 μL of the filtrate with 500 μL of *S. maltophilia* (OD600 ≈ 0.6) in 5 mL of LB medium, followed by shaking incubation at 37 °C overnight. After centrifugation at 12,000× *g* for 3 min, the resulting supernatant was subjected to filtration through a 0.22 μm pore-size membrane filter for the removal of residual bacterial cells and particulate debris. Subsequently, mixing 100 μL of the filtrate with 100 μL of bacterial culture in 5 mL of semi-solid LB top agar, poured evenly onto LB solid medium plates, and incubated at 37 °C overnight to obtain phage plaques. The phage was purified through three consecutive rounds of single-plaque isolation.

### 4.3. Transmission Electron Microscopy

For negative staining, phage particles were treated using 2% (*w*/*v*) phosphotungstic acid (pH 7.0; Beijing Solarbio Science & Technology Co., Ltd., Beijing, China) for a duration of 2 min, and their morphology was visualized using a JEM-1200EX transmission electron microscope (JEOL Ltd., Akishima, Tokyo, Japan).

### 4.4. Phage Host Range Analysis

Eighteen clinical *S. maltophilia* isolates, obtained from clinical settings, were provided by the hospital. The host range was determined by spotting 2.5 μL of phage suspensions (10^2^–10^8^ PFU/mL, prepared from an initial lysate of 1 × 10^8^ PFU/mL) onto lawns of various bacterial strains, with subsequent incubation at 37 °C for 8 h.

### 4.5. Thermal and pH Stability

To assess thermal stability, phage solutions were incubated at a series of temperatures (4 °C, 30 °C, 40 °C, 50 °C, 60 °C, 70 °C, 80 °C, and 90 °C) for 1 h. For pH stability assessment, phage solutions were incubated in phosphate-buffered saline (Beijing Solarbio Science & Technology Co., Ltd., Beijing, China) adjusted to across a pH gradient (pH 2–12) at 37 °C for 1 h.

### 4.6. One-Step Growth Curve Analysis and Determination of Optimal Infection Multiplicity (MOI)

Log-phase *S. maltophilia* host cells (OD600 ≈ 0.6) were mixed with the phage at different multiplicities of infection (MOIs; phage-to-bacteria ratios: 10, 1, 0.1, 0.01, 0.001). After incubation at 37 °C for 4 h, PFU were counted. The optimal MOI (typically achieving >90% of the initial host concentration in PFU counts) was selected for subsequent experiments. Host cells (OD600 ≈ 0.6) were adsorbed with the phage at the optimized MOI at 37 °C for 10 min. Free phages were discarded by centrifugation (12,000× *g*, 1 min) and washing, and the bacterial pellet was resuspended in pre-warmed fresh LB medium (t = 0). At intervals of 10–30 min over a period of up to 180 min, 1 mL aliquots were collected, serially diluted, and the titer of progeny phages was determined using the double-layer agar plaque assay. The phage growth curve was plotted as titer versus infection time.

### 4.7. Phage Whole-Genome Sequencing

To further investigate the genetic information of the phage, its genome was sent to Biomarker Technologies Co., Ltd. (Beijing, China) for sequencing. Illumina sequencing was performed as follows: DNA was first fragmented into 200–500 bp libraries using ultrasound; after adapter ligation, DNA fragments were randomly attached to the flowcell surface and amplified via bridge PCR to form DNA clusters, amplifying base signal intensity. Whole-genome sequencing was conducted using the paired-end sequencing-by-synthesis method for comprehensive and accurate coverage. High-throughput sequencing was performed on an Illumina platform. ORFs were predicted with Pharokka v1.2.4 [35] with default parameters. Hypothetical functions of the identified ORFs were inferred via BLASTp v2.17.0 [36] and HHpred (version: 57c8707149031cc9f8edceba362c71a3762bdbf8) [37]. Potential virulence and antibiotic resistance genes were screened using the Virulence Factor Database (VFDB) v4.0 [38] and ResFinder v4.7.2 [39], respectively. The XAN_XB1 genome map was visualized with Proksee v1.0.0a6 [40]. Amino acid sequences of the MCP and terminase large subunit were aligned with MAFFT v7.487 [41], and low-quality alignment sites were trimmed using trimAl v1.4 [42]. Phylogenetic trees were constructed in IQ-TREE v2.1.4 [43] and visualized and annotated using tvBOT v2.6.1 [44]. Intergenomic ANI between XAN_XB1 and related phages was calculated using VIRIDIC v1.1 [45] with default parameters, following ICTV species demarcation criteria.

### 4.8. Therapeutic Efficacy of Phage XAN_XB1 in a Mouse Model of S. Maltophilia Infection

An immunosuppressed mouse model of *S. maltophilia* infection was established following previously described methods [20]. In our study, a single colony of *Stenotrophomonas maltophilia* strain 200019 was inoculated into LB broth and incubated at 37 °C with shaking at 220 rpm for 8 h to reach the logarithmic growth phase. Briefly, log-phase bacteria were harvested, washed twice with saline, and resuspended at 12,000 g, 1 min to a concentration of 5 × 10^9^ CFU/mL. SPF male BALB/c mice (3–4 weeks old, 14–18 g) were obtained from the Experimental Animal Center (Xi’an, China). All animal procedures were approved by the Ethics Committee of Xi’an Jiaotong University (Approval No. XJTUAE2025-3849). Mice were housed under a 12:12 h light/dark cycle with ad libitum access to food and water. After a three-day acclimatization period, immunosuppression was induced to mimic infection in immunocompromised patients. As described previously, mice received an intraperitoneal injection of cyclophosphamide (200 mg/kg; MedChemExpress LLC, Monmouth Junction, NJ, USA) on day 4, followed by a combined intraperitoneal injection of cyclophosphamide (200 mg/kg) and dexamethasone (12.5 mg/kg; MedChemExpress LLC, NJ, USA) under isoflurane anesthesia on day 1 prior to infection.

Immunocompromised mice were randomly allocated into three groups (*n* = 16 per group). The phage-only control group received 40 μL of saline intranasally, followed by an intranasal administration of phages (2 × 10^9^ PFU/mouse) 6 h later. The infection control group was infected intranasally with a bacterial suspension (2 × 10^8^ CFU/mouse), followed by 40 μL of saline intranasally at 6 hpi. The treatment group was infected identically to the infection control group and received intranasal phage therapy (2 × 10^9^ PFU/mouse) at 6 hpi. Survival was monitored daily for 7 days in ten mice per group. At 30 h post-phage treatment, six randomly selected mice from each group were anesthetized via intraperitoneal injection of urethane (1.4 g/kg; Beijing Sinopharm Chemical Reagent Co., Ltd., Shanghai, China). Blood was collected for measurement of serum IL-6 and PCT levels. Lung tissues were harvested and divided: one portion was fixed in formalin for H&E staining (BaSo Technology Co., Ltd., Zhuhai, China) and histopathological evaluation, while the other was homogenized and plated for quantification of viable bacterial counts [46].

### 4.9. Statistical Analysis

Survival curves were derived via the Kaplan–Meier method, with comparative analyses conducted using the log-rank test. Quantitative data excluding survival endpoints were reported as mean ± standard deviation and analyzed with Student’s *t*-test. Differences were considered statistically significant when *p* < 0.05.

## 5. Conclusions

The XAN_XB1 is a novel lytic phage with strong lytic activity, broad host range, environmental stability, a safe genomic profile, and efficacy in mice models. These properties make it a promising candidate for therapeutic application against multidrug-resistant *S. maltophilia* infections. Future studies should evaluate its potential synergies with antibiotics or other phages, paving the way for its development into a viable clinical treatment.

## Data Availability

The genome sequence accession number is PX401838, additional experimental data and analytical details can be provided upon reasonable request.
